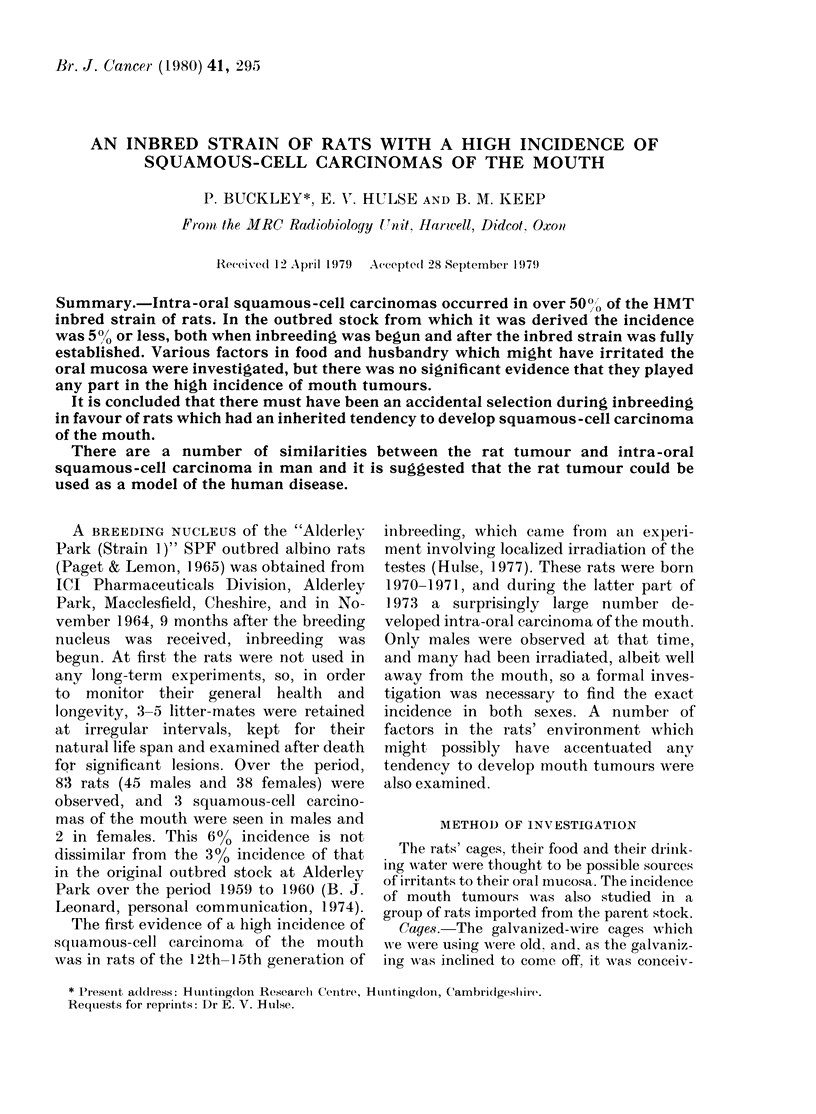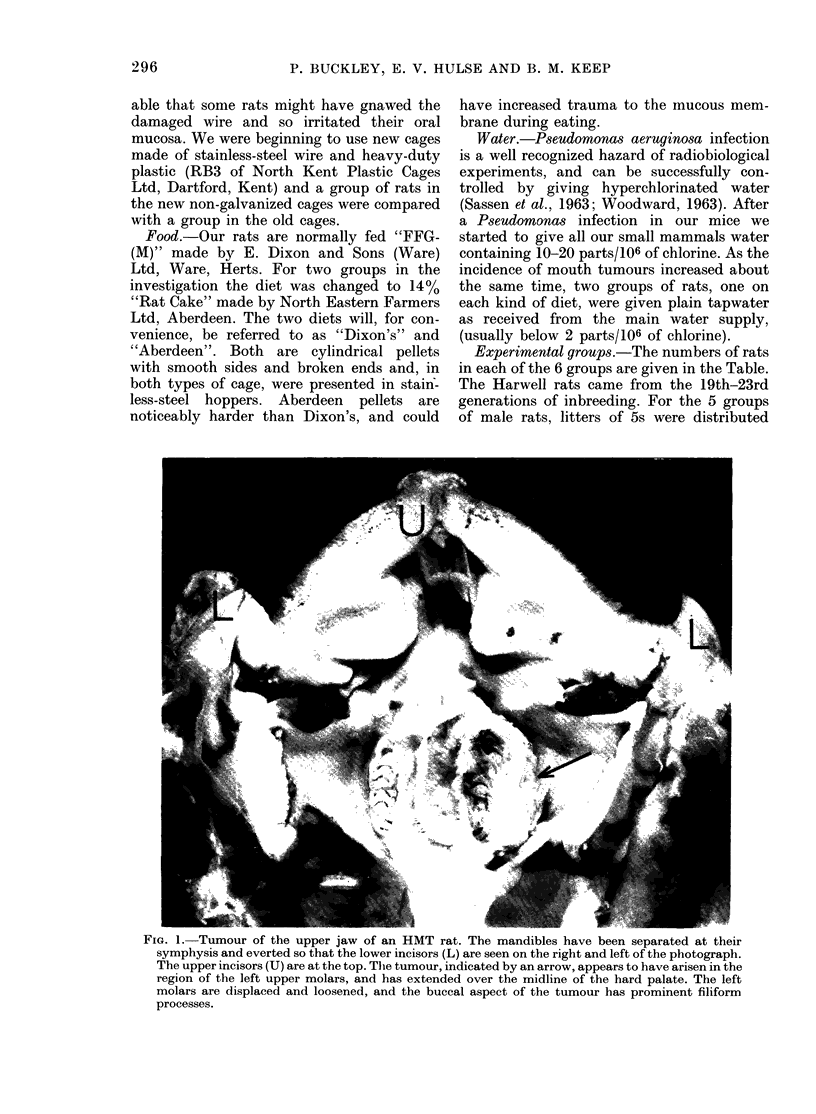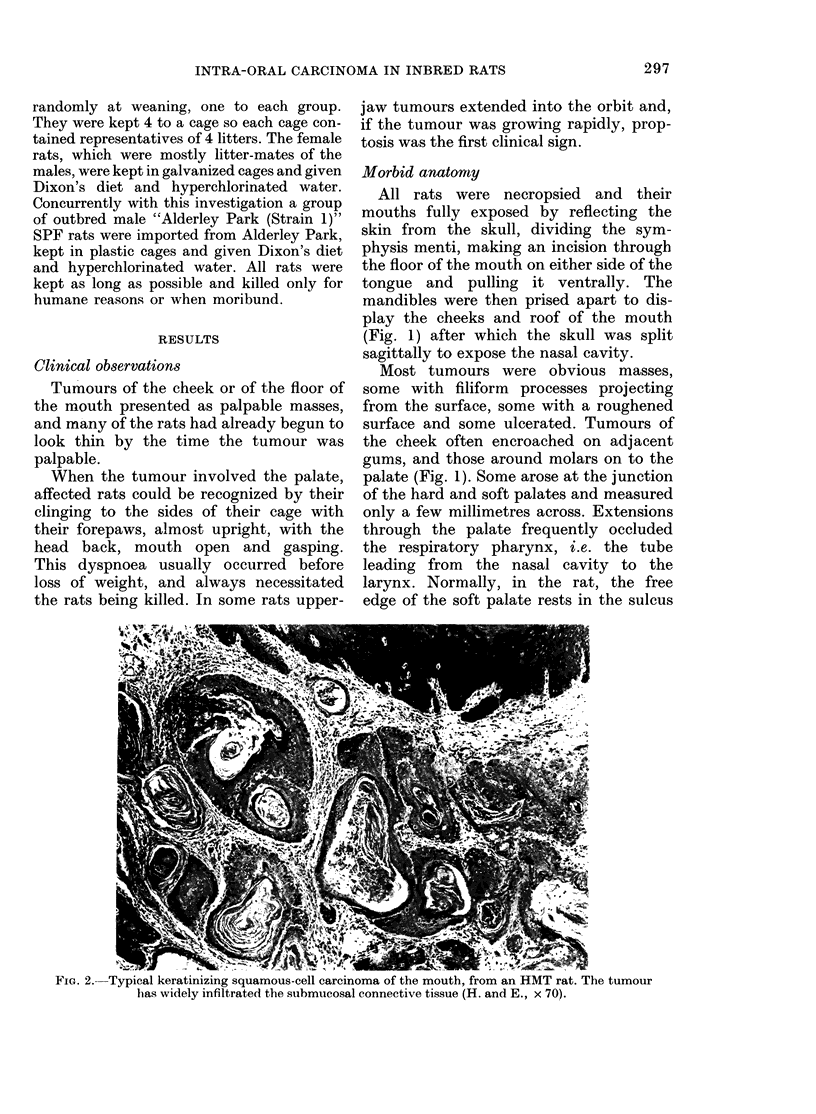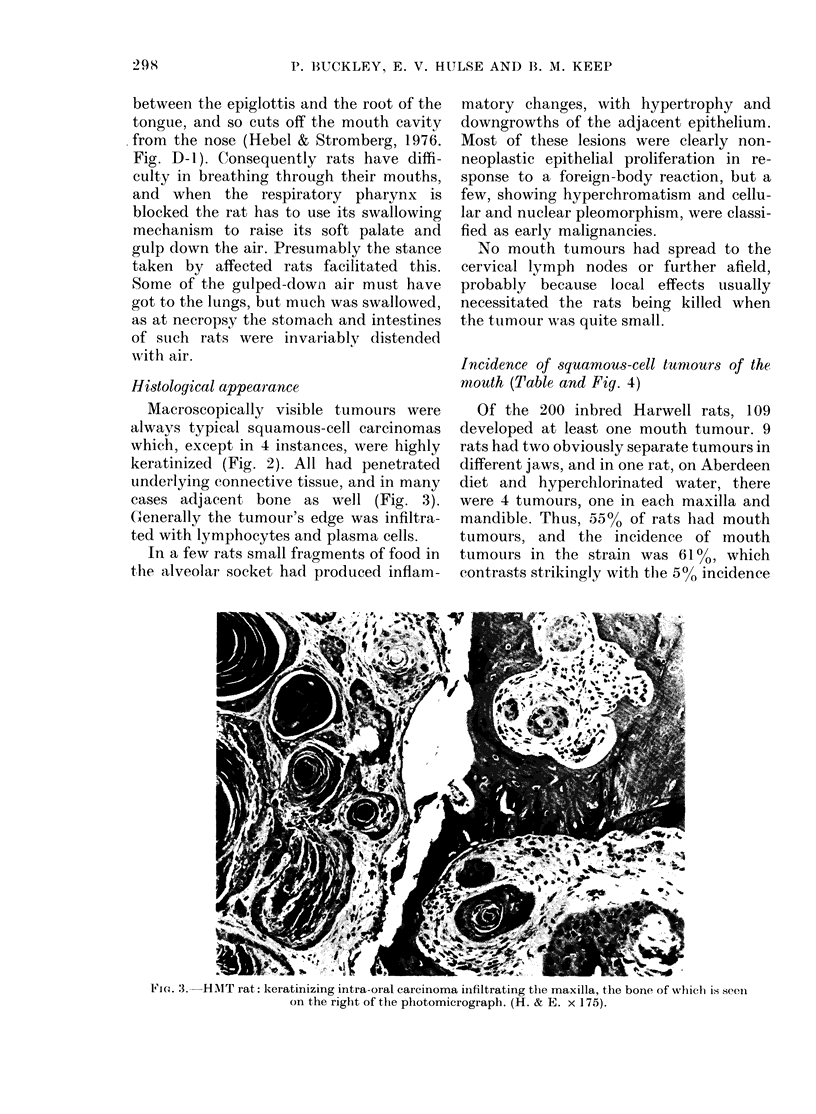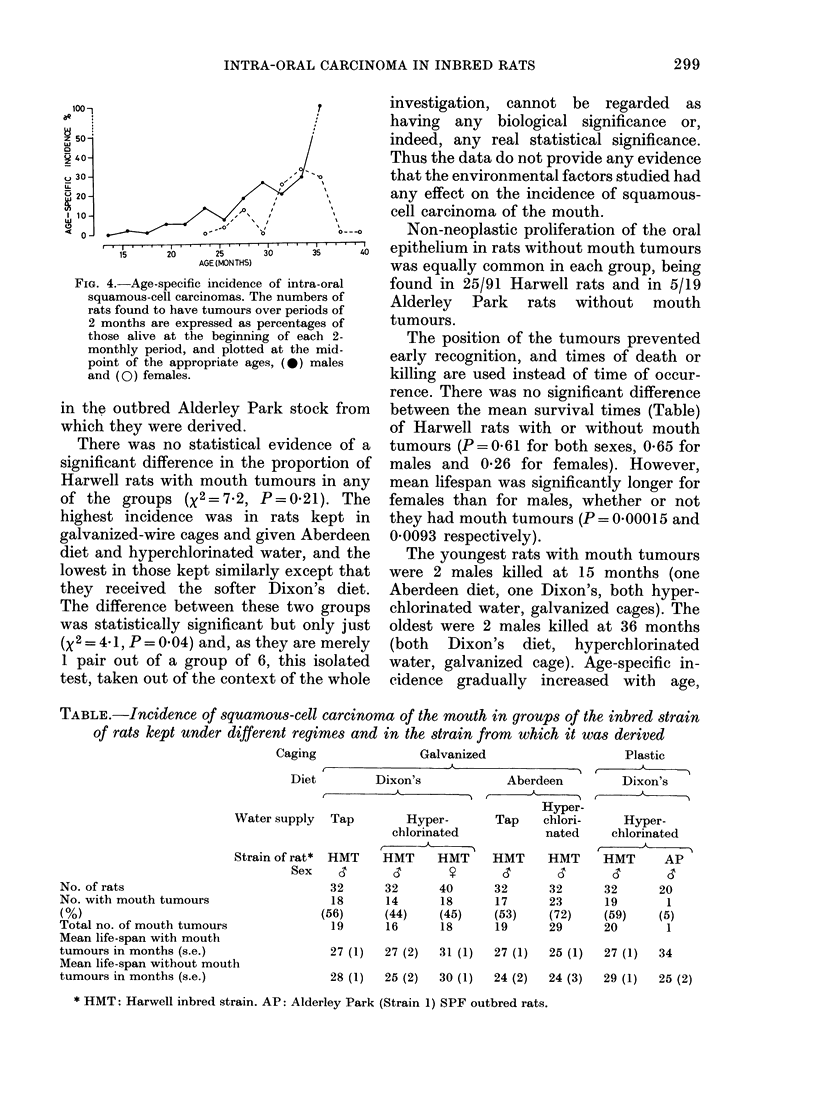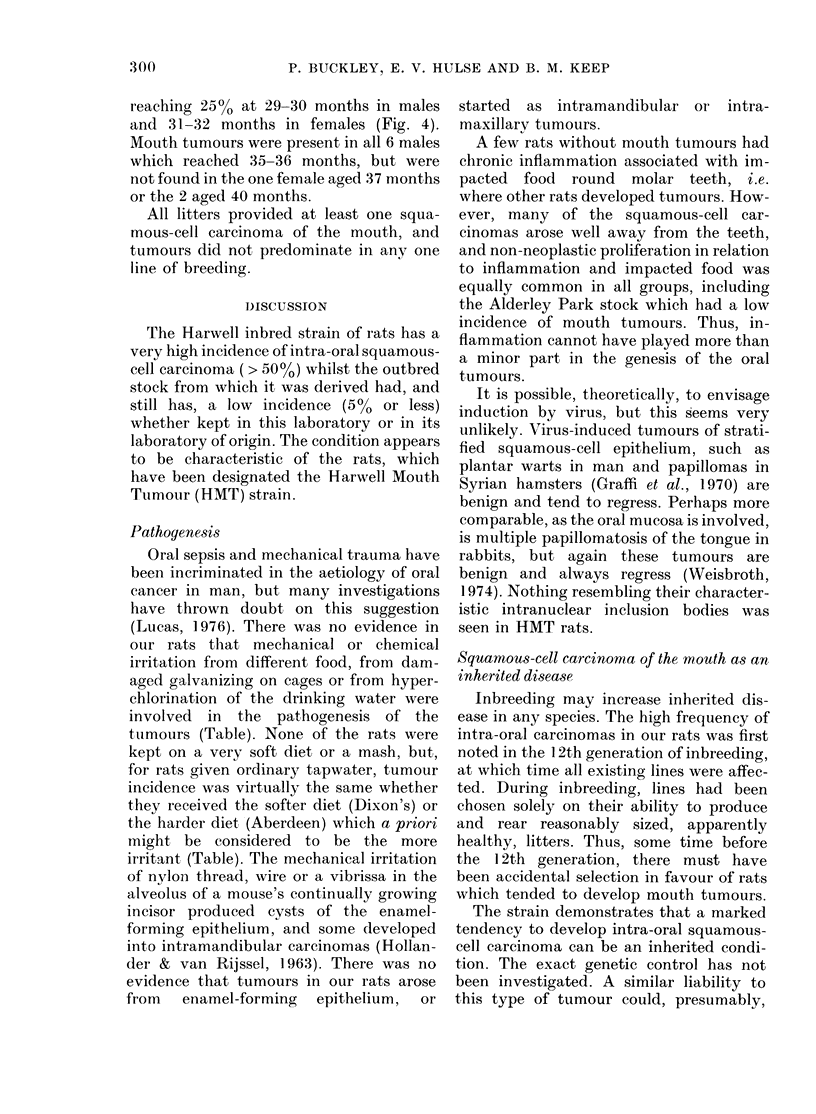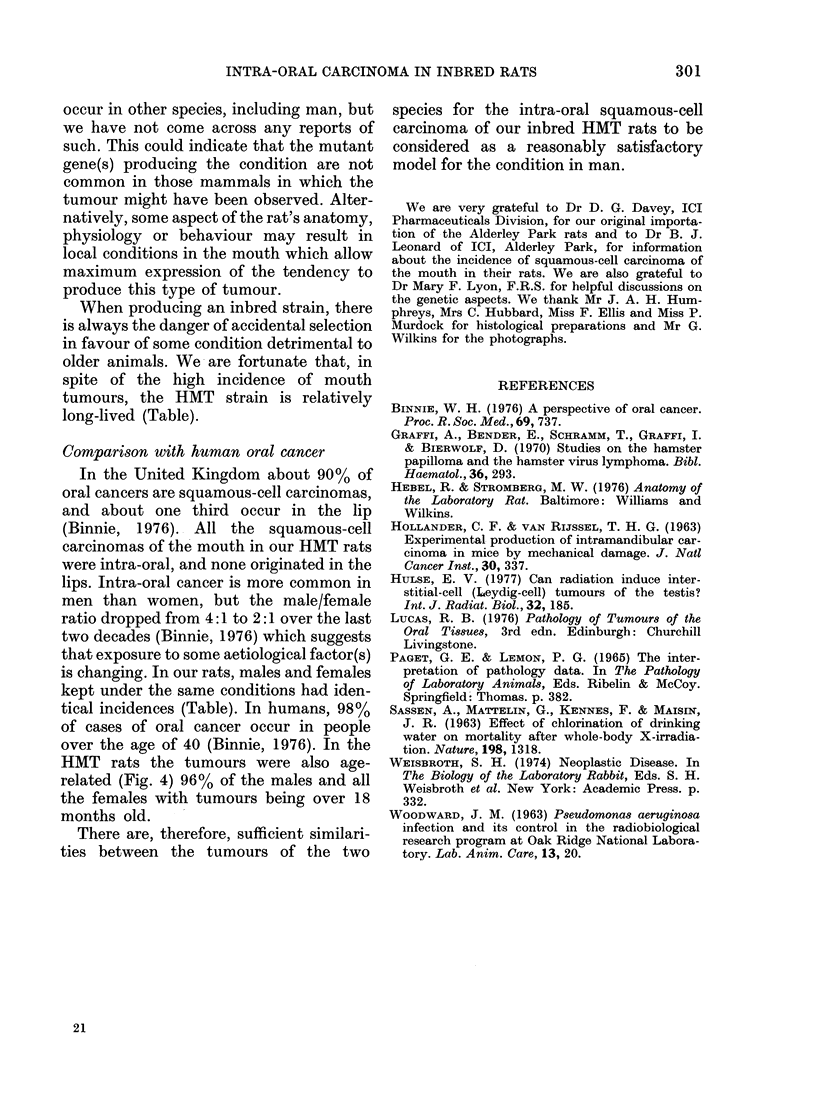# An inbred strain of rats with a high incidence of squamous-cell carcinomas of the mouth.

**DOI:** 10.1038/bjc.1980.42

**Published:** 1980-02

**Authors:** P. Buckley, E. V. Hulse, B. M. Keep

## Abstract

**Images:**


Br. J. Cancer (1980) 41, 295

### AN INBRED STRAIN OF RATS WITH A HIGH INCIDENCE OF SQUAMOUS-CELL CARCINOMAS OF THE MOUTH

P. BUCKLEY*, E. V. HULSE AND B. M. KEEP

*From the MRC Radiobiology Unit, Harwell, Didcot, Oxon*

Received 12 April 1979 Accepted 28 September 1979

**Summary.—Intra-oral squamous-cell carcinomas occurred in over 50% of the HMT inbred strain of rats. In the outbred stock from which it was derived the incidence was 5% or less, both when inbreeding was begun and after the inbred strain was fully established. Various factors in food and husbandry which might have irritated the oral mucosa were investigated, but there was no significant evidence that they played any part in the high incidence of mouth tumours.**

**It is concluded that there must have been an accidental selection during inbreeding in favour of rats which had an inherited tendency to develop squamous-cell carcinoma of the mouth.**

**There are a number of similarities between the rat tumour and intra-oral squamous-cell carcinoma in man and it is suggested that the rat tumour could be used as a model of the human disease.**

A BREEDING NUCLEUS of the "Alderley Park (Strain 1)" SPF outbred albino rats (Paget & Lemon, 1965) was obtained from ICI Pharmaceuticals Division, Alderley Park, Macclesfield, Cheshire, and in November 1964, 9 months after the breeding nucleus was received, inbreeding was begun. At first the rats were not used in any long-term experiments, so, in order to monitor their general health and longevity, 3–5 litter-mates were retained at irregular intervals, kept for their natural life span and examined after death for significant lesions. Over the period, 83 rats (45 males and 38 females) were observed, and 3 squamous-cell carcinomas of the mouth were seen in males and 2 in females. This 6% incidence is not dissimilar from the 3% incidence of that in the original outbred stock at Alderley Park over the period 1959 to 1960 (B. J. Leonard, personal communication, 1974).

The first evidence of a high incidence of squamous-cell carcinoma of the mouth was in rats of the 12th–15th generation of inbreeding, which came from an experiment involving localized irradiation of the testes (Hulse, 1977). These rats were born 1970–1971, and during the latter part of 1973 a surprisingly large number developed intra-oral carcinoma of the mouth. Only males were observed at that time, and many had been irradiated, albeit well away from the mouth, so a formal investigation was necessary to find the exact incidence in both sexes. A number of factors in the rats' environment which might possibly have accentuated any tendency to develop mouth tumours were also examined.

### METHOD OF INVESTIGATION

The rats' cages, their food and their drinking water were thought to be possible sources of irritants to their oral mucosa. The incidence of mouth tumours was also studied in a group of rats imported from the parent stock.

*Cages.*—The galvanized-wire cages which we were using were old, and, as the galvanizing was inclined to come off, it was conceiv-

* Present address: Huntingdon Research Centre, Huntingdon, Cambridgeshire.

Requests for reprints: Dr E. V. Hulse.

able that some rats might have gnawed the damaged wire and so irritated their oral mucosa. We were beginning to use new cages made of stainless-steel wire and heavy-duty plastic (RB3 of North Kent Plastic Cages Ltd, Dartford, Kent) and a group of rats in the new non-galvanized cages were compared with a group in the old cages.

*Food.*—Our rats are normally fed "FFG-(M)" made by E. Dixon and Sons (Ware) Ltd, Ware, Herts. For two groups in the investigation the diet was changed to 14% "Rat Cake" made by North Eastern Farmers Ltd, Aberdeen. The two diets will, for convenience, be referred to as "Dixon's" and "Aberdeen". Both are cylindrical pellets with smooth sides and broken ends and, in both types of cage, were presented in stainless-steel hoppers. Aberdeen pellets are noticeably harder than Dixon's, and could have increased trauma to the mucous membrane during eating.

*Water.*—*Pseudomonas aeruginosa* infection is a well recognized hazard of radiobiological experiments, and can be successfully controlled by giving hyperchlorinated water (Sassen *et al.*, 1963; Woodward, 1963). After a *Pseudomonas* infection in our mice we started to give all our small mammals water containing 10–20 parts/$10^6$ of chlorine. As the incidence of mouth tumours increased about the same time, two groups of rats, one on each kind of diet, were given plain tapwater as received from the main water supply, (usually below 2 parts/$10^6$ of chlorine).

*Experimental groups.*—The numbers of rats in each of the 6 groups are given in the Table. The Harwell rats came from the 19th–23rd generations of inbreeding. For the 5 groups of male rats, litters of 5s were distributed

FIG. 1.—Tumour of the upper jaw of an HMT rat. The mandibles have been separated at their symphysis and everted so that the lower incisors (L) are seen on the right and left of the photograph. The upper incisors (U) are at the top. The tumour, indicated by an arrow, appears to have arisen in the region of the left upper molars, and has extended over the midline of the hard palate. The left molars are displaced and loosened, and the buccal aspect of the tumour has prominent filiform processes.

randomly at weaning, one to each group. They were kept 4 to a cage so each cage contained representatives of 4 litters. The female rats, which were mostly litter-mates of the males, were kept in galvanized cages and given Dixon's diet and hyperchlorinated water. Concurrently with this investigation a group of outbred male "Alderley Park (Strain 1)" SPF rats were imported from Alderley Park, kept in plastic cages and given Dixon's diet and hyperchlorinated water. All rats were kept as long as possible and killed only for humane reasons or when moribund.

### RESULTS

### *Clinical observations*

Tumours of the cheek or of the floor of the mouth presented as palpable masses, and many of the rats had already begun to look thin by the time the tumour was palpable.

When the tumour involved the palate, affected rats could be recognized by their clinging to the sides of their cage with their forepaws, almost upright, with the head back, mouth open and gasping. This dyspnoea usually occurred before loss of weight, and always necessitated the rats being killed. In some rats upper-jaw tumours extended into the orbit and, if the tumour was growing rapidly, proptosis was the first clinical sign.

### *Morbid anatomy*

All rats were necropsied and their mouths fully exposed by reflecting the skin from the skull, dividing the symphysis menti, making an incision through the floor of the mouth on either side of the tongue and pulling it ventrally. The mandibles were then prised apart to display the cheeks and roof of the mouth (Fig. 1) after which the skull was split sagittally to expose the nasal cavity.

Most tumours were obvious masses, some with filiform processes projecting from the surface, some with a roughened surface and some ulcerated. Tumours of the cheek often encroached on adjacent gums, and those around molars on to the palate (Fig. 1). Some arose at the junction of the hard and soft palates and measured only a few millimetres across. Extensions through the palate frequently occluded the respiratory pharynx, *i.e.* the tube leading from the nasal cavity to the larynx. Normally, in the rat, the free edge of the soft palate rests in the sulcus

FIG. 2.—Typical keratinizing squamous-cell carcinoma of the mouth, from an HMT rat. The tumour has widely infiltrated the submucosal connective tissue (H. and E., × 70).

between the epiglottis and the root of the tongue, and so cuts off the mouth cavity from the nose (Hebel & Stromberg, 1976. Fig. D-1). Consequently rats have difficulty in breathing through their mouths, and when the respiratory pharynx is blocked the rat has to use its swallowing mechanism to raise its soft palate and gulp down the air. Presumably the stance taken by affected rats facilitated this. Some of the gulped-down air must have got to the lungs, but much was swallowed, as at necropsy the stomach and intestines of such rats were invariably distended with air.

*Histological appearance*

Macroscopically visible tumours were always typical squamous-cell carcinomas which, except in 4 instances, were highly keratinized (Fig. 2). All had penetrated underlying connective tissue, and in many cases adjacent bone as well (Fig. 3). Generally the tumour's edge was infiltrated with lymphocytes and plasma cells.

In a few rats small fragments of food in the alveolar socket had produced inflammatory changes, with hypertrophy and downgrowths of the adjacent epithelium. Most of these lesions were clearly non-neoplastic epithelial proliferation in response to a foreign-body reaction, but a few, showing hyperchromatism and cellular and nuclear pleomorphism, were classified as early malignancies.

No mouth tumours had spread to the cervical lymph nodes or further afield, probably because local effects usually necessitated the rats being killed when the tumour was quite small.

*Incidence of squamous-cell tumours of the mouth (Table and Fig. 4)*

Of the 200 inbred Harwell rats, 109 developed at least one mouth tumour. 9 rats had two obviously separate tumours in different jaws, and in one rat, on Aberdeen diet and hyperchlorinated water, there were 4 tumours, one in each maxilla and mandible. Thus, 55% of rats had mouth tumours, and the incidence of mouth tumours in the strain was 61%, which contrasts strikingly with the 5% incidence

FIG. 3.—HMT rat: keratinizing intra-oral carcinoma infiltrating the maxilla, the bone of which is seen on the right of the photomicrograph. (H. & E. × 175).

FIG. 4.—Age-specific incidence of intra-oral squamous-cell carcinomas. The numbers of rats found to have tumours over periods of 2 months are expressed as percentages of those alive at the beginning of each 2-monthly period, and plotted at the mid-point of the appropriate ages, (●) males and (○) females.

in the outbred Alderley Park stock from which they were derived.

There was no statistical evidence of a significant difference in the proportion of Harwell rats with mouth tumours in any of the groups ($\chi^2 = 7{\cdot}2$, $P = 0{\cdot}21$). The highest incidence was in rats kept in galvanized-wire cages and given Aberdeen diet and hyperchlorinated water, and the lowest in those kept similarly except that they received the softer Dixon's diet. The difference between these two groups was statistically significant but only just ($\chi^2 = 4{\cdot}1$, $P = 0{\cdot}04$) and, as they are merely 1 pair out of a group of 6, this isolated test, taken out of the context of the whole investigation, cannot be regarded as having any biological significance or, indeed, any real statistical significance. Thus the data do not provide any evidence that the environmental factors studied had any effect on the incidence of squamous-cell carcinoma of the mouth.

Non-neoplastic proliferation of the oral epithelium in rats without mouth tumours was equally common in each group, being found in 25/91 Harwell rats and in 5/19 Alderley Park rats without mouth tumours.

The position of the tumours prevented early recognition, and times of death or killing are used instead of time of occurrence. There was no significant difference between the mean survival times (Table) of Harwell rats with or without mouth tumours ($P = 0{\cdot}61$ for both sexes, 0·65 for males and 0·26 for females). However, mean lifespan was significantly longer for females than for males, whether or not they had mouth tumours ($P = 0{\cdot}00015$ and 0·0093 respectively).

The youngest rats with mouth tumours were 2 males killed at 15 months (one Aberdeen diet, one Dixon's, both hyperchlorinated water, galvanized cages). The oldest were 2 males killed at 36 months (both Dixon's diet, hyperchlorinated water, galvanized cage). Age-specific incidence gradually increased with age,

TABLE.—*Incidence of squamous-cell carcinoma of the mouth in groups of the inbred strain of rats kept under different regimes and in the strain from which it was derived*

| Caging | Galvanized | | | | | Plastic | |
|---|---|---|---|---|---|---|---|
| Diet | Dixon's | | | Aberdeen | | Dixon's | |
| Water supply | Tap | Hyper-chlorinated | | Tap | Hyper-chlorinated | Hyper-chlorinated | |
| Strain of rat* | HMT | HMT | HMT | HMT | HMT | HMT | AP |
| Sex | ♂ | ♂ | ♀ | ♂ | ♂ | ♂ | ♂ |
| No. of rats | 32 | 32 | 40 | 32 | 32 | 32 | 20 |
| No. with mouth tumours | 18 | 14 | 18 | 17 | 23 | 19 | 1 |
| (%) | (56) | (44) | (45) | (53) | (72) | (59) | (5) |
| Total no. of mouth tumours | 19 | 16 | 18 | 19 | 29 | 20 | 1 |
| Mean life-span with mouth tumours in months (s.e.) | 27 (1) | 27 (2) | 31 (1) | 27 (1) | 25 (1) | 27 (1) | 34 |
| Mean life-span without mouth tumours in months (s.e.) | 28 (1) | 25 (2) | 30 (1) | 24 (2) | 24 (3) | 29 (1) | 25 (2) |

* HMT: Harwell inbred strain. AP: Alderley Park (Strain 1) SPF outbred rats.

reaching 25% at 29–30 months in males and 31–32 months in females (Fig. 4). Mouth tumours were present in all 6 males which reached 35–36 months, but were not found in the one female aged 37 months or the 2 aged 40 months.

All litters provided at least one squamous-cell carcinoma of the mouth, and tumours did not predominate in any one line of breeding.

### DISCUSSION

The Harwell inbred strain of rats has a very high incidence of intra-oral squamous-cell carcinoma ( > 50%) whilst the outbred stock from which it was derived had, and still has, a low incidence (5% or less) whether kept in this laboratory or in its laboratory of origin. The condition appears to be characteristic of the rats, which have been designated the Harwell Mouth Tumour (HMT) strain.

### *Pathogenesis*

Oral sepsis and mechanical trauma have been incriminated in the aetiology of oral cancer in man, but many investigations have thrown doubt on this suggestion (Lucas, 1976). There was no evidence in our rats that mechanical or chemical irritation from different food, from damaged galvanizing on cages or from hyperchlorination of the drinking water were involved in the pathogenesis of the tumours (Table). None of the rats were kept on a very soft diet or a mash, but, for rats given ordinary tapwater, tumour incidence was virtually the same whether they received the softer diet (Dixon's) or the harder diet (Aberdeen) which *a priori* might be considered to be the more irritant (Table). The mechanical irritation of nylon thread, wire or a vibrissa in the alveolus of a mouse's continually growing incisor produced cysts of the enamel-forming epithelium, and some developed into intramandibular carcinomas (Hollander & van Rijssel, 1963). There was no evidence that tumours in our rats arose from enamel-forming epithelium, or started as intramandibular or intramaxillary tumours.

A few rats without mouth tumours had chronic inflammation associated with impacted food round molar teeth, *i.e.* where other rats developed tumours. However, many of the squamous-cell carcinomas arose well away from the teeth, and non-neoplastic proliferation in relation to inflammation and impacted food was equally common in all groups, including the Alderley Park stock which had a low incidence of mouth tumours. Thus, inflammation cannot have played more than a minor part in the genesis of the oral tumours.

It is possible, theoretically, to envisage induction by virus, but this seems very unlikely. Virus-induced tumours of stratified squamous-cell epithelium, such as plantar warts in man and papillomas in Syrian hamsters (Graffi *et al.*, 1970) are benign and tend to regress. Perhaps more comparable, as the oral mucosa is involved, is multiple papillomatosis of the tongue in rabbits, but again these tumours are benign and always regress (Weisbroth, 1974). Nothing resembling their characteristic intranuclear inclusion bodies was seen in HMT rats.

### *Squamous-cell carcinoma of the mouth as an inherited disease*

Inbreeding may increase inherited disease in any species. The high frequency of intra-oral carcinomas in our rats was first noted in the 12th generation of inbreeding, at which time all existing lines were affected. During inbreeding, lines had been chosen solely on their ability to produce and rear reasonably sized, apparently healthy, litters. Thus, some time before the 12th generation, there must have been accidental selection in favour of rats which tended to develop mouth tumours.

The strain demonstrates that a marked tendency to develop intra-oral squamous-cell carcinoma can be an inherited condition. The exact genetic control has not been investigated. A similar liability to this type of tumour could, presumably, occur in other species, including man, but we have not come across any reports of such. This could indicate that the mutant gene(s) producing the condition are not common in those mammals in which the tumour might have been observed. Alternatively, some aspect of the rat's anatomy, physiology or behaviour may result in local conditions in the mouth which allow maximum expression of the tendency to produce this type of tumour.

When producing an inbred strain, there is always the danger of accidental selection in favour of some condition detrimental to older animals. We are fortunate that, in spite of the high incidence of mouth tumours, the HMT strain is relatively long-lived (Table).

*Comparison with human oral cancer*

In the United Kingdom about 90% of oral cancers are squamous-cell carcinomas, and about one third occur in the lip (Binnie, 1976). All the squamous-cell carcinomas of the mouth in our HMT rats were intra-oral, and none originated in the lips. Intra-oral cancer is more common in men than women, but the male/female ratio dropped from 4:1 to 2:1 over the last two decades (Binnie, 1976) which suggests that exposure to some aetiological factor(s) is changing. In our rats, males and females kept under the same conditions had identical incidences (Table). In humans, 98% of cases of oral cancer occur in people over the age of 40 (Binnie, 1976). In the HMT rats the tumours were also age-related (Fig. 4) 96% of the males and all the females with tumours being over 18 months old.

There are, therefore, sufficient similarities between the tumours of the two species for the intra-oral squamous-cell carcinoma of our inbred HMT rats to be considered as a reasonably satisfactory model for the condition in man.

We are very grateful to Dr D. G. Davey, ICI Pharmaceuticals Division, for our original importation of the Alderley Park rats and to Dr B. J. Leonard of ICI, Alderley Park, for information about the incidence of squamous-cell carcinoma of the mouth in their rats. We are also grateful to Dr Mary F. Lyon, F.R.S. for helpful discussions on the genetic aspects. We thank Mr J. A. H. Humphreys, Mrs C. Hubbard, Miss F. Ellis and Miss P. Murdock for histological preparations and Mr G. Wilkins for the photographs.

### REFERENCES

BINNIE, W. H. (1976) A perspective of oral cancer. *Proc. R. Soc. Med.*, **69**, 737.

GRAFFI, A., BENDER, E., SCHRAMM, T., GRAFFI, I. & BIERWOLF, D. (1970) Studies on the hamster papilloma and the hamster virus lymphoma. *Bibl. Haematol.*, **36**, 293.

HEBEL, R. & STROMBERG, M. W. (1976) *Anatomy of the Laboratory Rat.* Baltimore: Williams and Wilkins.

HOLLANDER, C. F. & VAN RIJSSEL, T. H. G. (1963) Experimental production of intramandibular carcinoma in mice by mechanical damage. *J. Natl Cancer Inst.*, **30**, 337.

HULSE, E. V. (1977) Can radiation induce interstitial-cell (Leydig-cell) tumours of the testis? *Int. J. Radiat. Biol.*, **32**, 185.

LUCAS, R. B. (1976) *Pathology of Tumours of the Oral Tissues*, 3rd edn. Edinburgh: Churchill Livingstone.

PAGET, G. E. & LEMON, P. G. (1965) The interpretation of pathology data. In *The Pathology of Laboratory Animals*, Eds. Ribelin & McCoy. Springfield: Thomas. p. 382.

SASSEN, A., MATTELIN, G., KENNES, F. & MAISIN, J. R. (1963) Effect of chlorination of drinking water on mortality after whole-body X-irradiation. *Nature*, **198**, 1318.

WEISBROTH, S. H. (1974) Neoplastic Disease. In *The Biology of the Laboratory Rabbit*, Eds. S. H. Weisbroth *et al.* New York: Academic Press. p. 332.

WOODWARD, J. M. (1963) *Pseudomonas aeruginosa* infection and its control in the radiobiological research program at Oak Ridge National Laboratory. *Lab. Anim. Care*, **13**, 20.